# Solution structure of the second bromodomain of Brd2 and its specific interaction with acetylated histone tails

**DOI:** 10.1186/1472-6807-7-57

**Published:** 2007-09-12

**Authors:** Hongda Huang, Jiahai Zhang, Weiqun Shen, Xingsheng Wang, Jiawen Wu, Jihui Wu, Yunyu Shi

**Affiliations:** 1 Hefei National Laboratory for Physical Sciences at Microscale and School of Life Science, University of Science and Technology of China, Hefei, Anhui 230026, People's Republic of China

## Abstract

**Background:**

Brd2 is a transcriptional regulator and belongs to BET family, a less characterized novel class of bromodomain-containing proteins. Brd2 contains two tandem bromodomains (BD1 and BD2, 46% sequence identity) in the N-terminus and a conserved motif named ET (extra C-terminal) domain at the C-terminus that is also present in some other bromodomain proteins. The two bromodomains have been shown to bind the acetylated histone H4 and to be responsible for mitotic retention on chromosomes, which is probably a distinctive feature of BET family proteins. Although the crystal structure of Brd2 BD1 is reported, no structure features have been characterized for Brd2 BD2 and its interaction with acetylated histones.

**Results:**

Here we report the solution structure of human Brd2 BD2 determined by NMR. Although the overall fold resembles the bromodomains from other proteins, significant differences can be found in loop regions, especially in the ZA loop in which a two amino acids insertion is involved in an uncommon *π*-helix, termed *π*D. The helix *π*D forms a portion of the acetyl-lysine binding site, which could be a structural characteristic of Brd2 BD2 and other BET bromodomains. Unlike Brd2 BD1, BD2 is monomeric in solution. With NMR perturbation studies, we have mapped the H4-AcK12 peptide binding interface on Brd2 BD2 and shown that the binding was with low affinity (2.9 mM) and in fast exchange. Using NMR and mutational analysis, we identified several residues important for the Brd2 BD2-H4-AcK12 peptide interaction and probed the potential mechanism for the specific recognition of acetylated histone codes by Brd2 BD2.

**Conclusion:**

Brd2 BD2 is monomeric in solution and dynamically interacts with H4-AcK12. The additional secondary elements in the long ZA loop may be a common characteristic of BET bromodomains. Surrounding the ligand-binding cavity, five aspartate residues form a negatively charged collar that serves as a secondary binding site for H4-AcK12. We suggest that Brd2 BD1 and BD2 may possess distinctive roles and cooperate to regulate Brd2 functions. The structure basis of Brd2 BD2 will help to further characterize the functions of Brd2 and its BET members.

## Background

Eukaryotic transcription is a highly regulated process. Transcription activation in eukaryotes requires the modification of histones on enhancers and promoters [1,2]. Histone acetylation plays a major role in this modification, and forms histone codes in combination with methylation, phosphorylation and ubiquitination [3-5]. One potential postulate of histone codes is that defined patterns of modification in histones can be recognized by specific factors. Bromodomain, a conserved ~110 amino acid module that is originally identified by sequence alignment as a common motif among *Drosophila *Brahma, female-sterile homeotic (fsh) and four other potential transcription regulators [6-8], can recognize acetylation of histones [9-13] and is thought to participate in deciphering the histone codes [3,14-17]. Bromodomain-containing proteins have been classified into several distinct subgroups: (i) histone acetyltransferases (HATs), including GCN5, P/CAF and TAF_II_250; (ii) ATP-dependent chromatin-remodeling complexes, including Brahma, Swi2, Snf2 and Brg1; (iii) the less-characterized BET (bromodomain and extra C-terminal domain) family, which is a novel class of transcriptional regulators carrying two tandem bromodomains and an ET domain [18]. Retention on chromosomes during mitosis is likely a distinctive feature of the BET family [19-21], because proteins of other bromodomain families are displaced from chromosomes during mitosis [22,23]. There are four mammalian protein members of BET family: Brd2/Ring3 (also referred to as Fsrg1), ORFX (Fsrg2), Brd4/HUNK1 (Fsrg4), Brdt (Fsrg3).

Brd2 gene is initially identified as an open reading frame localized to the class II major histocompatibility locus on human chromosome 6p21.3 and is related to the *Drosophila *gene *female sterile homeotic *(*fsh*) [24]. The homologous genes in mouse [25], frog [26], and zebrafish [27], have been reported. Brd2 is a nuclear-localized serine-threonine kinase that has elevated activity in human leukemias [28,29]. It has been shown to synergistically transactivate cell cycle regulatory genes, including *cyclin D1, cyclin A, cyclin E *and *dihydrofolate reductase *(*dhf*), in combination with Ras or MEKK through E2Fs [30]. A murine homologue of Brd2 is contained in a mediator transcription complex that involves homologues of the yeast transcriptional regulators Med6, Med7, Rgr1, and Srb7 [31]. Later on, Brd2 is reported to associate with a transcription complex containing E2F-1, E2F-2, TBP and chromatin remodeling machines [30,32,33], and to serve as a bridge between E2F-1 and TBP [34]. Brd2 is also reported to associate with mediator subunits cyclin-dependent kinase 8 (Cdk8), thyroid receptor-associated protein 220 (TRAP220), and the Pol II large subunit (Pol II ls) [35]. Directed overexpression of Brd2 in mouse lymphocytes is found to develop B-cell lymphoma and leukemia [36]. In addition, Brd2 can interact with latent nuclear antigen (LANA) of Kaposi's sarcoma-associated herpesvirus (KSHV/HHV-8) [37]. Taken together, these reports indicate that Brd2 can serve as a transcription regulator and a contributing element in oncogenesis.

The bromodomains of Brd2 have been shown to selectively bind to histone H4 tails acetylated at Lys12 (AcK12) and to be responsible for mitotic retention on chromosomes [21]. The bromodomains are essential for transcriptional activation of important E2F-responsive genes by Brd2 through E2Fs. Bromodomain-deletion derivant of Brd2 has a high level of autophosphorylation activity and exhibites significant E2F-1 phosphorylation *in vitro *compared with wild-type [32]. It is also reported that the association of Brd2 with E2Fs, mediator components and Pol II is dependent on the presence of and is stimulated by acetylated histone H3 and H4 peptides [35]. The association with E2Fs and specific interaction with acetylated histones both establish anchor points for Brd2-containing transcription complex to *cyclin A *promoter [33]. In addition, Brd4 (another BET member) also associates with mitotic chromosomes, and tethers viral DNA to host's mitotic chromosomes [20,38]. Brd4 interacts with P-TEFb through its bromodomains and stimulates Pol II-dependent transcription [39,40]. Hence the bromodomains and their recognition of specific acetylated histones play important roles in functions of BET family members. Bromodomains of the BET family differ substantially from those of other families in amino acid sequence, despite that they share conserved hydrophobic residues in the hydrophobic core of the domain. Previous studies showed that different bromodomains have different specificities for binding to acetylated histones in living cells [21]. However, it is yet unknown the structural basis for Brd2 recognizing the specific acetylated histone code. Here, we have determined the solution structure of the second bromodomain (BD2) of Brd2 by NMR and studied its binding properties to acetylated histone H4. Comparing this structure with bromodomains from other proteins, significant differences are found in loop regions; specifically some additional secondary structures exist in Brd2 BD2. Using NMR titration experiments, the conserved hydrophobic cavity that is previously characterized in other bromodomains is found to serve as a binding site for histone H4 acetylated at K12. Through mutational analysis, we detected that the basic residues flanking the acetylated lysine of histone H4 peptides, and some resides in loop regions of Brd2 BD2 are important for their mutual recognition. Our results provide further structural and functional details aimed at revealing how Brd2 and other BET members recognize specific acetylated histone codes and thus impact transcription.

## Results

### Structure description

**Figure 1:**
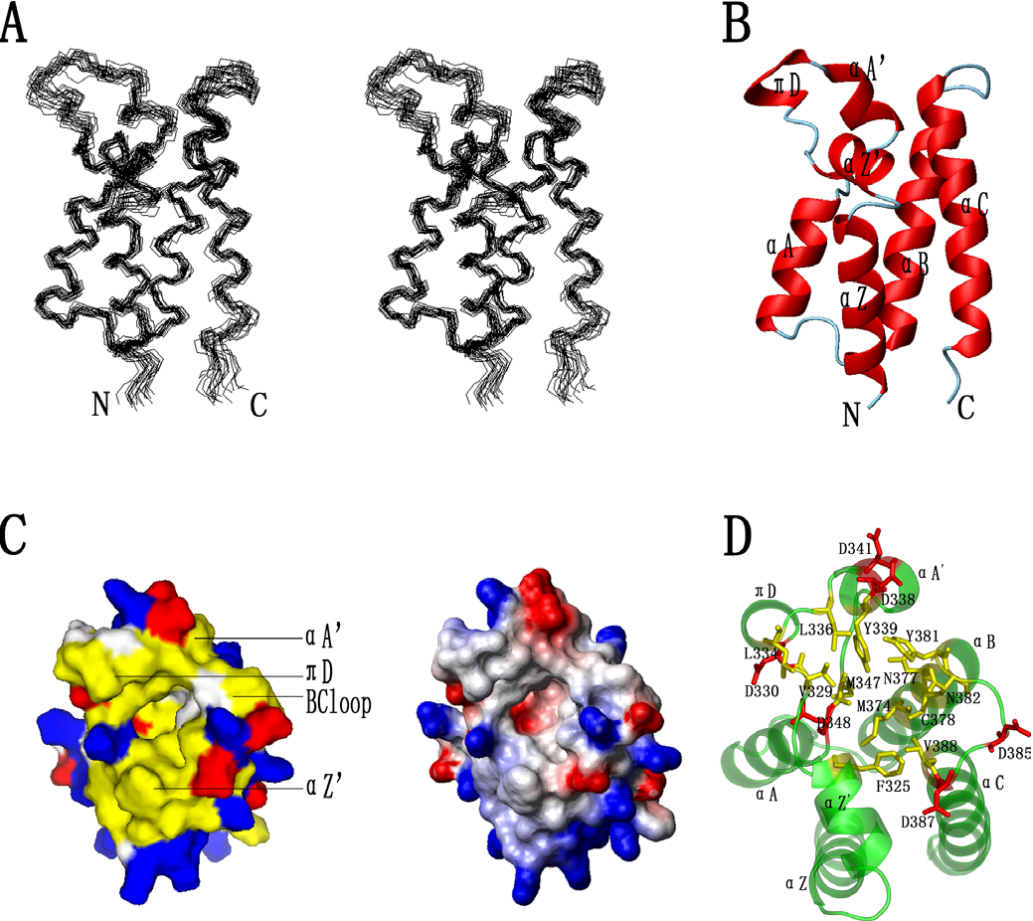
**Structure and molecular contact surfaces of Brd2 BD2**. (**A**) Stereoview of the selected 20 structures of Brd2 BD2, superimposed on backbone atoms (N, C_*α *_and C'). (**B**) Ribbon representation of the average, energy-minimized structure with the secondary structure elements highlighted. The helix nomenclature follows that of hsP/CAF bromodomain [9]. (**C**) Contact surface emphasized surface hydrophobic potential (left) and surface electrostatic potential (right) at the acetyl-lysine binding site. Yellow denotes hydrophobic potential; red negative potential; and blue positive potential. (**D**) A clear view of showing the conserved or type conserved side chains lined the hydrophobic cavity, and denoting the negative-charged collar formed by residues D330, D338, D341, D385 and D387. A, B, C and D were produced with MOLMOL or PyMOL.

Table 1 lists the structural statistics for the 20 structures deposited in the Protein Data Bank [PDB: 2G4A]. A large, negative Lennard-Jones potential energy was observed (-360.86 ± 15.49 kcal mol^-1^), indicating good nonbonded geometry of the structure. For the region from residue Q302 to M406, the RMSD values to the mean structure were 0.69Å for backbone atoms and 1.18 Å for all heavy atoms [see Additional file 1]. The RMSD values for the well-defined secondary structure elements were 0.58 and 1.07, respectively. In the ZA and BC loops, there was an increasing level of disorder compared with the rigid secondary elements [see Additional file 1]. A stereoview of the backbone superimposition of the final 20 structures are shown in Figure 1A.

**Table 1:** Structural statistics for the selected 20 structures of Brd2 BD2^a^

Number of NMR restraints used in the structure calculation	
Intra-residue	563
Sequential (|i-j| = 1)	573
Medium-range (|i-j|<5)	475
Long-range (|i-j|≥5)	355
Total NOE restraints	1966
Hydrogen bonds	49
Dihedral angle restraints	146
Lennard-Jones potential energy (kcal mol-1)	-360.86 ± 15.49
RMSD from idealized covalent geometry	
Bonds (Å)	0.0010 ± 0.00002
Angles (deg)	0.2878 ± 0.0016
Impropers (deg)	0.0972 ± 0.0038
RMSD from experimental restraints	
Distance (Å)	0.0037 ± 0.0005
cdih (deg)	0.0436 ± 0.0214
Coordinate RMSD from mean (Å) residues 302 to 406	
All backbone atoms	0.69
All heavy atoms	1.18
Secondary structure elements^b^	
All backbone atoms	0.58
All heavy atoms	1.07
Ramachandran plot (% residues)	
Residues in most favored regions	87.0
Residues in additional allowed regions	10.3
Residues in generously allowed regions	2.5
Residues in disallowed regions	0.2

a None of the structure exhibits distance violations greater than 0.2 Å or dihedral angle violations greater than 2°.

b Residues Q302-S315, A319-Y326, H337-I342, L349-N358, A364-Y381, D387-K405.

**Figure 2:**
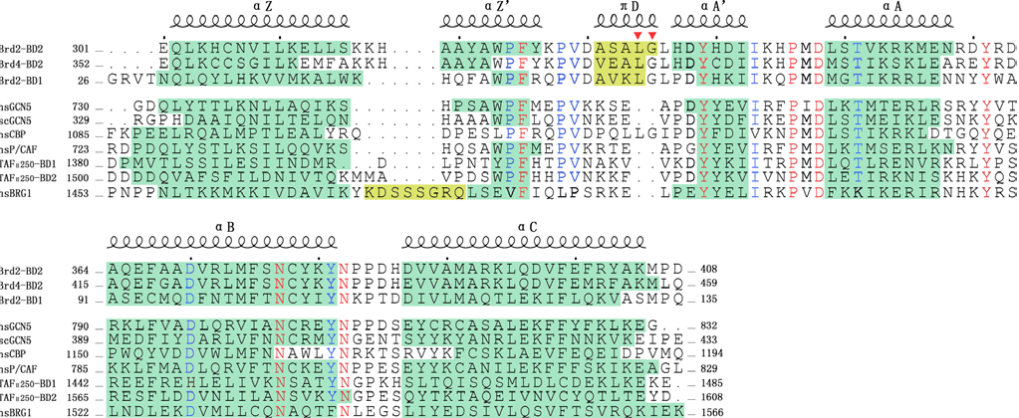
**Sequence alignment of Brd2 BD2**. Sequence alignment of Brd2 BD2 with a selected number of Bromodomains including Brd2 BD1, Brd4 BD2, hsGCN5, scGCN5, hsCBP, hsP/CAF, hsBRG1 and the two components from TAF_II_250. The sequences were aligned based on the experimentally determined three-dimensional structures of these bromodomains, highlighted in green. The secondary structure of Brd2 BD2 is indicated above the alignment. Residues identical in all sequences are shown in red and residues conserved are coloured in blue and residues corresponding to *β*Z sheet (hsBRG1) and helix *π*D are represented in yellow. The two amino acids insertion is indicated by triangle symbols (▼).

The tertiary structure of Brd2 BD2 consists of seven distinct helices and is totally 74% helical, which is consistent with the circular dichroism prediction (data not shown). Four of the *α*-helices, *α*Z (residues 302–315), *α*A (residues 349–358), *α*B (residues 364–381) and *α*C (residues 387–405), are arranged in closely compact, left-handed bundle (Figure 1B) in an antiparallel manner. Crossing angles between adjacent helices *α*Z and *α*A, *α*Z and *α*C are ~30°, while those between helices *α*A and *α*B, *α*B and *α*C are ~10–20°. Most of the slowly exchanging NH protons in ^1^H-^2^H exchange experiments were found within these regions [see Additional file 2], which indicated that protection occurred most likely by formation of hydrogen bonds here. In the long ZA loop between helices *α*Z and *α*A, there are three additional short helices: *α*-helices *α*Z' (residues 318–326) and *α*A' (residues 337–342), and an uncommon *π*-helix, termed helix *π*D (residue 331–335). The two helices *α*Z' and *α*A' are conserved in hsBRG1 and GCN5 bromodomains, while helix *π*D is also found in Brd2 BD1 [PDB: 1X0J] [41] and Brd4 BD2 [PDB: 2I8N]. The three helix structures were also confirmed by helix-typical NOEs. Besides, helix *α*Z' was further affirmed by ^1^H-^2^H exchange experiments. The helix *α*Z' is orthogonal to helices *α*Z and *α*A. Two hydrogen bonds are formed between backbone carbonyl groups of F325 and Y326 with backbone amide groups of L349 and S350, respectively, serving to cap the helix *α*A and help to anchor the long ZA loop. A typical 'capping box' motif in N cap [42] involving a hydrogen bond between OD1 of Asp (D348) and NH of N3 (T351) was also found to cap the helix *α*A. Both D348 and T351 are highly conserved in bromodomain sequences (Figure 2). Both helices *α*A' and *π*D may be quite flexible or in 'structure breath' states for ligand binding because helix *α*A' contacts only with helix *α*B (Figure 1B), helix *π*D has no residue interacting directly with the other helices, and few long-range NOEs and no slow-exchange NH protons are observed in these regions [43].

At the top of the protein (oriented as in Figure 1A), a groove with a cavity at the bottom could be seen clearly on the contact surface (Figure 1C). The cavity is lined by the side-chains of residues (F325, V329, L334, L336, Y339, M347, D348, M374, N377, C378, Y381, N382 and V388) mainly from the ZA and BC loops (Figure 1D). Most of the residues are hydrophobic and highly conserved in bromodomain sequences. From surface hydrophobic potential and electrostatic potential mapping, the cavity is shown to be hydrophobic and electroneutral in nature, suitable for hydrophobic contact mediating protein-peptide interactions. Five aspartate residues (D330, D338, D341, D385 and D387) form a negatively charged collar surrounding the edge of the hydrophobic cavity (Figure 1C and 1D). It has been proposed that these acidic residues may play a role in the specificity of bromodomain ligand binding [11].

### Structure comparison with other bromodomains

**Figure 3:**
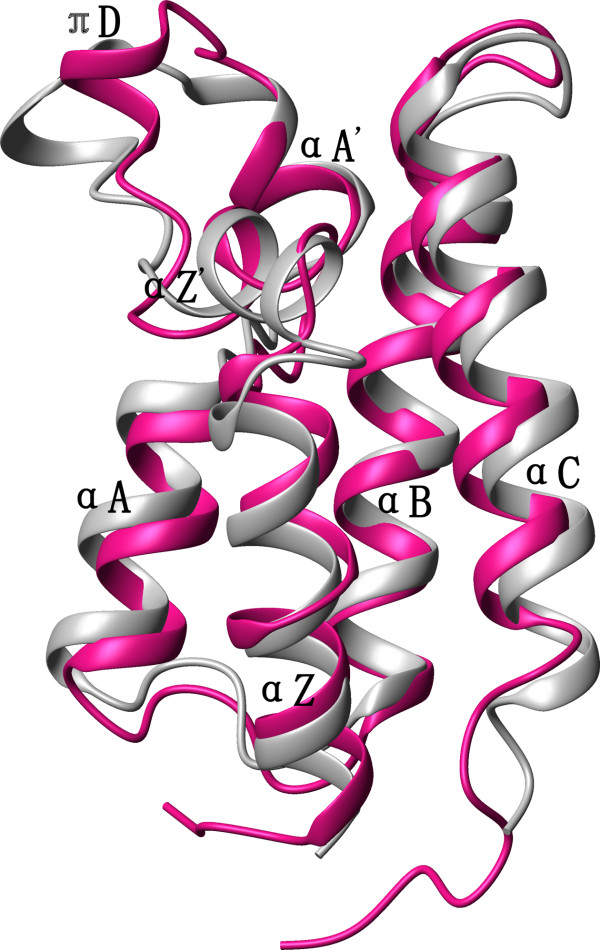
**Backbone superposition of Brd2 BD2 and BD1 structures**. Backbone superposition of the average, energy-minimized structure of Brd2 BD2 (grey) with the crystal structure of Brd2 BD1 (pink) [PDB: 1X0J]. The figure was generated with MOLMOL.

In spite that Brd2 BD2 share only 22–37% sequence identity with published bromodomain structures of hsGCN5, scGCN5, hsCBP, hsP/CAF, hsBRG1, and of the double bromodomain from TAF_II_250 [9,11-13,44-47], the overall secondary elements and structural fold of Brd2 BD2 resemble those published bromodomains, as shown by the backbone superposition of those structures on the average, energy-minimized structure of Brd2 BD2 (Figure 2) and [see Additional file 3]. The pairwise backbone RMSDs of those structures to Brd2 BD2 are between 1.6 to 2.6 Å. Recently, the structures of Brd2 BD1 and Brd4 BD2 (our lab unpublished data) have also been solved. Brd2 BD2 possesses high sequence identity and structural similarities to its BET members (Figure 2), with pairwise backbone RMSDs between 1.4 to 1.8 Å. Figure 3 shows the backbone superposition of Brd2 BD2 on Brd2 BD1. Our results give further evidence that the unique structural fold, especially the four-helix left-handed bundle involving helices *α*Z, *α*A, *α*B and *α*C, is highly conserved in the bromodomain family.

The majority of structural deviations are localized in the loop regions, particularly the long ZA loop. There are different additional secondary structures in the ZA loop, including helices *α*Z', *π*D and *α*A' (Figure 2). All the bromodomain structures solved possess helix *α*A', and all have helix *α*Z' except hsCBP. In the bromodomains of hsGCN5, hsBRG1 and Brd2 BD1 the helix *α*Z' is a 3_10_-helix [11,41,46], while in Brd2 BD2 it is an *α*-helix defined by typical *α*-helical NOE and hydrogen bond patterns [see Additional file 2]. Another character of helix *α*Z' in Brd2 BD2 is that it is longer compared with the others. An uncommon *π*-helix, helix *π*D, is localized in the center of the ZA loop of Brd2 BD2; relevant *α*-helices are present in Brd2 BD1 and Brd4 BD2, but no corresponding helices are present in other bromodomain structures (Figure 2). A sequence feature of Brd2 BD2 and its BET family members is that they have a long ZA loop and a two amino acids insertion. Coincidently, the two amino acids insertion of Brd2 BD2 (L334G335) is in the helix *π*D and contributes to the hydrophobic groove, consequently it may play a role in ligand selectivity. The helix *π*D could be a structural characteristic of Brd2 BD2 and other BET bromodomains (Figure 2 and Figure 3). In the sequence of hsBRG1 bromodomain, there is also an insertion which forms a *β*Z sheet between helices *α*Z and *α*Z'[46]. Both the uncommon helix *π*D in Brd2 BD2 and the *β*Z sheet in hsBRG1 support the view that bromodomains present in different proteins must harbor sufficient structural diversities to support specific recognition of acetylation codes.

### Binding interface of H4-AcK12 peptide on Brd2 BD2

**Figure 4:**
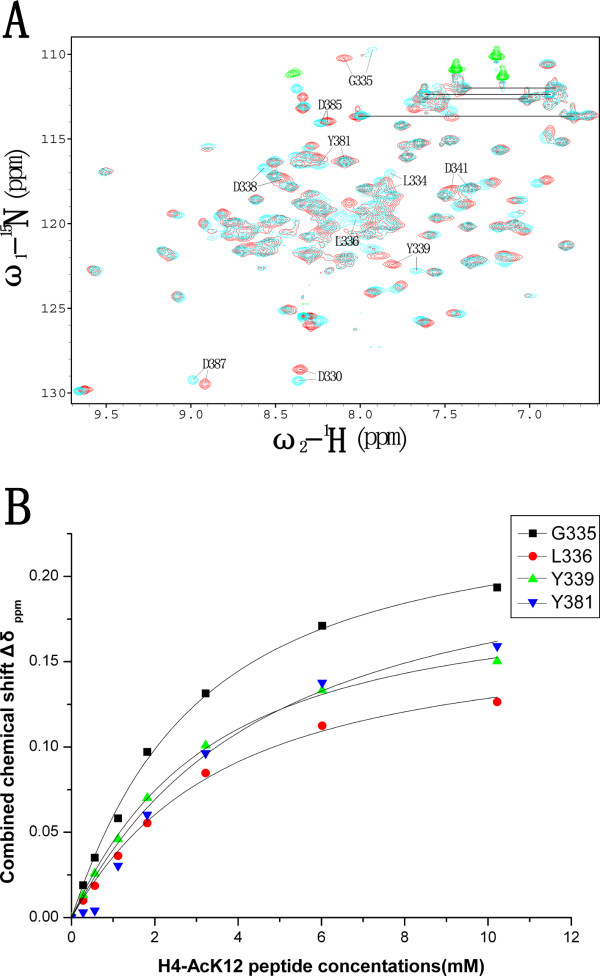
**Analysis of the interaction of Brd2 BD2 with H4-AcK12 peptide by NMR**. (**A**) Overlay of the ^15^N-HSQC spectra of Brd2 BD2 in free of H4-AcK12 peptide (red) and in the presence of H4-AcK12 peptide (cyan). Five of the most perturbed residues (L334, G335, L336, Y339, and Y381) and the five negative-charged aspartate residues (D330, D338, D341, D385 and D387) were denoted. (**B**) Binding constant was determined by monitoring the combined chemical shift perturbations (Δ*δ*_*ppm*_) of four most perturbed and not severely-overlapped residues (G335, L336, Y339 and Y381) as a function of concentrations of H4-AcK12 peptide.

A previous study has demonstrated that Brd2 selectively interacts with acetylated Lys 12 on histone H4 [21]. Accordingly, we have designed three histone tail peptides (Table 2), H2B-AcK5, H4-AcK8 and H4-AcK12, to titrate Brd2 BD2 using NMR experiments. The H4-AcK12 peptide induced a number of distinct chemical shift perturbations in the resonances of backbone amide groups of Brd2 BD2 in the ^1^H-^15^N HSQC spectrum (Figure 4). These chemical shift changes increased gradually throughout the titration that was taken to a maximum H4-AcK12 peptide: Brd2 BD2 ratio of 10:1. These data indicate interactions between Brd2 BD2 and H4-AcK12 peptide, and show that the peptide and the protein are in fast exchange between free and bound states on the NMR chemical shift timescale. Four most perturbed and not severely overlapped residues, G335, L336, Y339 and Y381, were plotted in figure 4B, and the dissociation constant (K_D_) for H4-AcK12 peptide was estimated to be ~2.9 mM [48]. Unlike H4-AcK12 peptide, H4-AcK8 peptide just slightly perturbed the chemical shifts of several residues such as G335, L336 and Y339 at very high peptide concentrations and didn't appear to interact significantly with Brd2 BD2. Addition of H2B AcK5 peptide resulted in little, if any, chemical shift perturbations of any protein residue, even at high concentrations. Totally, our titration analysis showed that Brd2 BD2 selectively bound to H4-AcK12, which was consistent with a previous report [21]; further we acquired the K_D _value for Brd2 BD2 binding to H4-AcK12 and determined this interaction to be dynamic in solution.

**Table 2:** Peptides used for binding to Brd2 BD2

Name	Sequence	Derivation
**H2B-AcK5**	SDPA-AcK-SAPAPKK	residues 1–12 of histone H2B
**H4-AcK8**	SGRGKGG-AcK-GLGK	residues 1–12 of histone H4
**H4-AcK12**	GKGLG-AcK-GGAKR	residues 7–17 of histone H4
**H4-L10A**	GKGAG-AcK-GGAKR	Leu 10 to Ala mutation of H4-AcK12
**H4-L10G**	GKGGG-AcK-GGAKR	Leu 10 to Gly mutation of H4-AcK12
**H4-A15G**	GKGLG-AcK-GGGKR	Ala 15 to Gly mutation of H4-AcK12
**H4-acid**	GDGLG-AcK-GGADE	Lys 8 to Asp, Lys 16 to Asp and Arg 17 to Glu mutations of H4-AcK12
**H4-U**	GKGLGKGGAKR	residues 7–17 of histone H4

**Figure 5:**
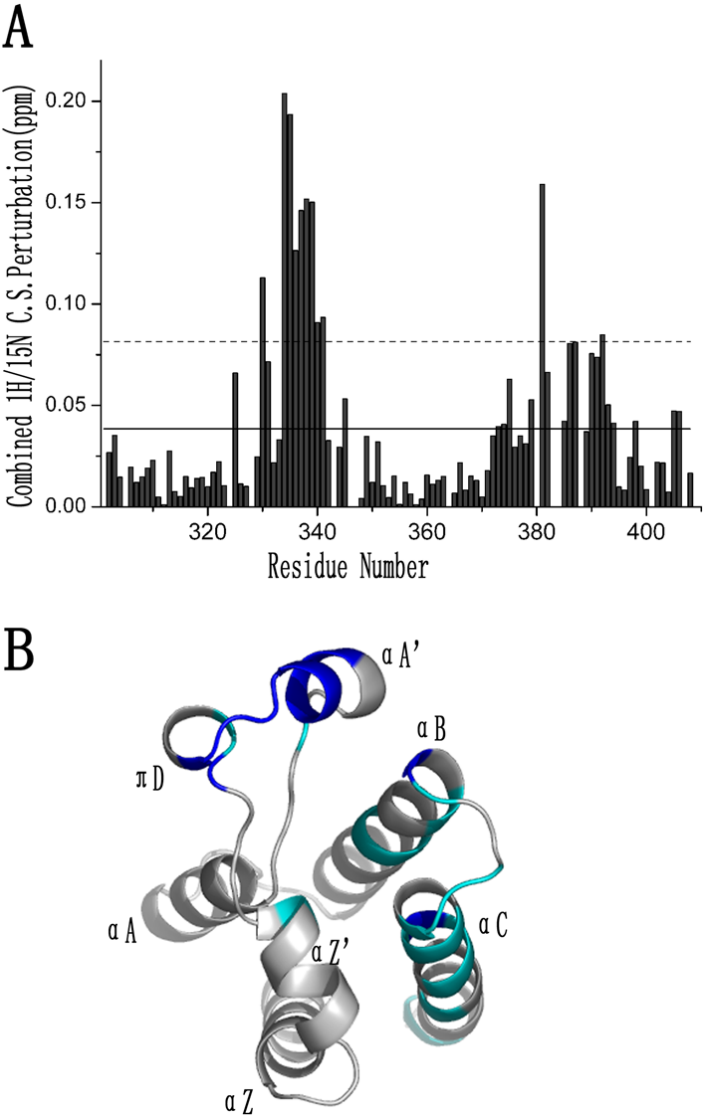
**Binding interface of H4-AcK12 peptide on Brd2 BD2**. (**A**) A histogram view of combined chemical shift perturbations of Brd2 BD2 by addition of H4-AcK12 peptide. The mean value is denoted as a solid line and the mean value plus one standard deviation as a dash line. (**B**) A ribbon diagram view mapping the binding interface of H4-AcK12 peptide on Brd2 BD2. The residues, whose combined chemical shift changes were more than the mean value plus one standard deviation and above the mean value, are colored in blue and cyan respectively. The figure B was generated in PyMOL.

Additionally, our titration results made us to move forward to map the binding interface of H4-AcK12 peptide on Brd2 BD2 (Figure 5). Most residues experienced significant chemical shift changes upon addition of H4-AcK12 peptide were concentrated in the ZA and BC loop, and the *α*-helical regions immediately flanking the BC loop. Among these residues, D330, L334, G335, L336, H337, D338, Y339, H340, D341, Y381 and A392 showed combined chemical shift changes of more than the mean value plus one standard deviation, and residues F325, A331, H345, L373, M374, F375, Y379, N382, D385, H386, D387, A390, M391, R393, K394, V398, K405 and M406 had combined chemical shift changes larger than mean value. Many of the residues that were significantly disturbed contribute to the hydrophobic pocket of Brd2 BD2 that serves as a primary binding surface for acetyl-lysine as in the other bromodomains (Figure 1D) [9,11-13,44-47].

### Mutational analysis

**Figure 6:**
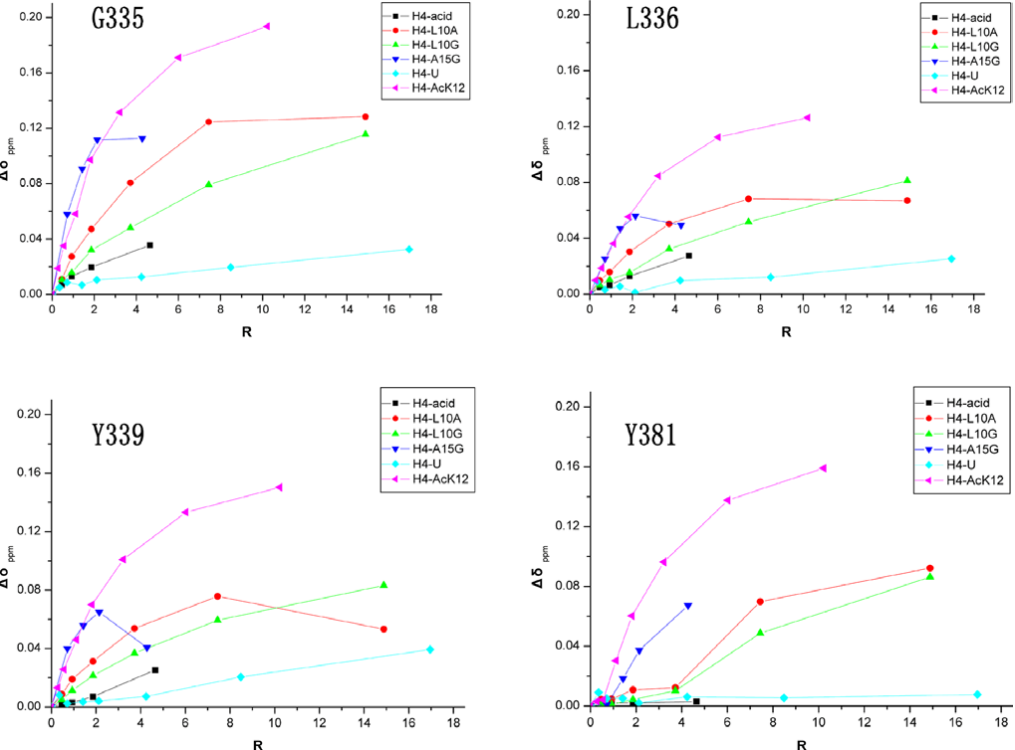
**NMR titrations of Brd2 BD2 with mutated histone H4 tails**. Brd2 BD2 was titrated with different H4-AcK12 peptide mutants, including H4-L10A, H4-L10G, H4-A15G, H4-acid and the unacetylated peptide H4-U. Combined chemical shift perturbation was calculated using the equation, Δδppm=(ΔδHN)2+(ΔδNαN)2
 MathType@MTEF@5@5@+=feaafiart1ev1aaatCvAUfKttLearuWrP9MDH5MBPbIqV92AaeXatLxBI9gBaebbnrfifHhDYfgasaacH8akY=wiFfYdH8Gipec8Eeeu0xXdbba9frFj0=OqFfea0dXdd9vqai=hGuQ8kuc9pgc9s8qqaq=dirpe0xb9q8qiLsFr0=vr0=vr0dc8meaabaqaciaacaGaaeqabaqabeGadaaakeaacqqHuoariiGacqWF0oazdaWgaaWcbaGaemiCaaNaemiCaaNaemyBa0gabeaakiabg2da9maakaaabaGaeiikaGIaeuiLdqKae8hTdq2aaSbaaSqaaiabdIeaijabd6eaobqabaGccqGGPaqkdaahaaWcbeqaaiabikdaYaaakiabgUcaRiabcIcaOiabfs5aejab=r7aKnaaBaaaleaacqWGobGtaeqaaOGae8xSde2aaSbaaSqaaiabd6eaobqabaGccqGGPaqkdaahaaWcbeqaaiabikdaYaaaaeqaaaaa@489D@, and R = [peptide]/[bromodomain].

The complex structure of scGCN5 bromodomain with H4-AcK16 peptide revealed that the major binding determinant was the acetylated lysine and that the secondary interaction came from the side chains at K+2 and K+3 positions [13]. The scGCN5 bromodomain only contacted with acetylated Lys 16, His 18 (K+2) and Arg 19 (K+3). The corresponding residues in H4-AcK12 peptide are unconserved Gly 14 (K+2) and Ala 15 (K+3) (Table 2). We designed a set of mutations of the H4-AcK12 peptide to determine which residues are critical for binding Brd2 BD2. NMR titrations of wild type Brd2 BD2 were performed using H4-AcK12 mutant peptides, including H4-L10A, H4-L10G, H4-A15G, H4-acid and the unacetylated peptide H4-U (Table 2 and Figure 6). The H4-U and H4-acid peptides did not disturb any residue amide resonances of Brd2 BD2, indicating that acetylated H4 tail was the preferred substrate and that those basic residues, Lys 8, Lys 16 and Arg 17 of H4 tail, could play an important role in the interactions. The H4-L10A and H4-L10G peptides induced small but still significant resonance shifts. The chemical shift changes induced by H4-A15G were like those in response to H4-AcK12 at a low peptide: Brd2 BD2 ratio of less than 4:1. Strikingly, when H4-A15G peptide: Brd2 BD2 ratio was large than 4:1, all the amide resonances of Brd2 BD2 disappeared. This phenomenon was confirmed by repeated titration experiments with two different protein concentrations (data not shown).

**Figure 7:**
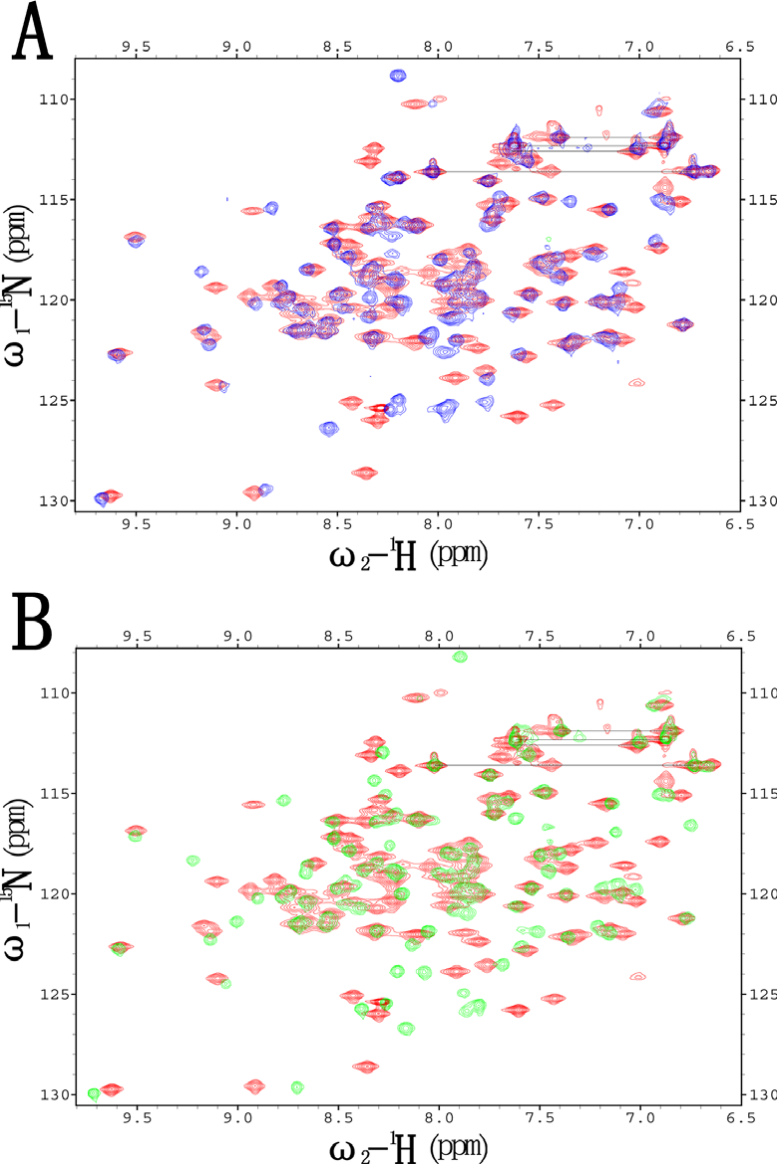
**Structural integrity analysis of Brd2 BD2 mutants**. The integrity of Brd2 BD2 mutants were assessed by their ^15^N-HSQC spectra. (**A**) Overlay of the ^15^N-HSQC spectra of Brd2 BD2 (red) and V329A (blue); (**B**) Overlay of the ^15^N-HSQC spectra of Brd2 BD2 (red) and N382A (green).

**Figure 8:**
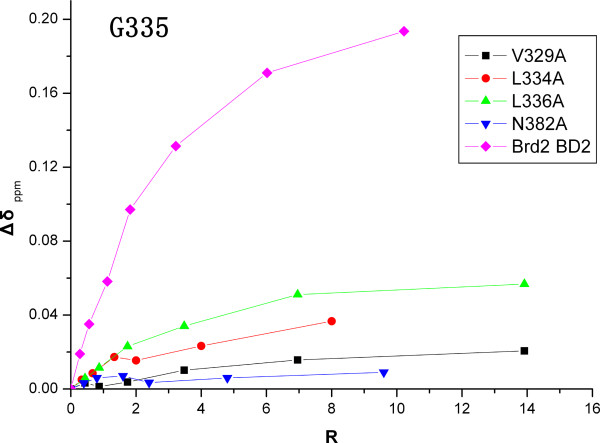
**Plot of Brd2 BD2 mutants titrations with H4-AcK12 peptide**. Brd2 BD2 mutants including V329A, L334A, L336A and N382A, were titrated with H4-AcK12 peptide. The doubtless and more significant perturbed residue G335 during titrations was followed.

Further more, Brd2 BD2 mutants, including V329A, L334A, L336A and N382A, were titrated with the H4-AcK12 peptide, respectively. The integrity of Brd2 BD2 mutants were assessed by their ^15^N-HSQC spectra. Compared with the ^15^N-HSQC spectrum of Brd2 BD2, mutants V329A and N382A showed significant differences of amide resonances for residues in the ZA and BC loop, and some residues localized in the termini of helices *α*B and *α*C; mutant L336A exhibited moderate differences for residues in the ZA and BC loop; whereas mutant L334A just slightly affected vicinal residues (Figure 7) and [see Additional file 4]. Both mutants V329A and N382A showed no binding to H4-AcK12 peptide, which should be attributed to severe disruption of the conformation of the binding cavity. Mutants L334A and L336A have fewer residues perturbed upon H4-AcK12 binding than wild type Brd2 BD2, and the perturbations are smaller. The unambiguous and significantly perturbed residue G335 was followed during titrations and plotted in figure 8.

### Dynamic properties of Brd2 BD2

**Figure 9:**
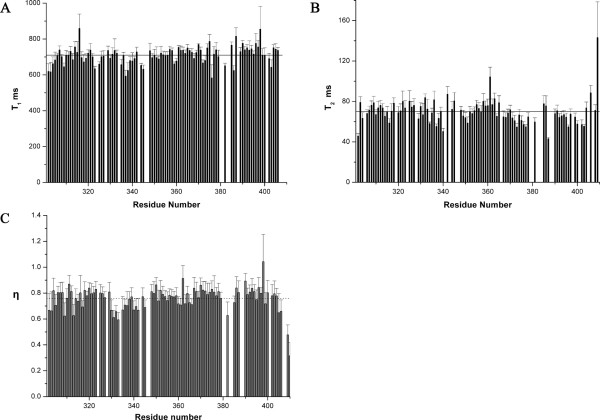
**Plot of the longitudinal and transverse relaxation times and ratio of intensity of NOE**. The longitudinal (T_1 _ms) (**A**) and transverse relaxation times (T_2 _ms) (**B**) and ratio of intensity of NOE (**C**) are plotted as a function of residue number of Brd2 BD2. Only those residues of which ^1^H-^15^N cross-peaks are resolved enough to permit accurate measurements of their intensities are included.

The longitudinal relaxation times (T_1 _ms), transverse relaxation times (T_2 _ms) and ratio of NOE intensity are recorded to probe the dynamic properties of Brd2 BD2. The T_2 _relaxation times were obtained for 85% of the backbone amide protons; the T_1 _relaxation times and ^1^H-^15^N heteronuclear NOE values were obtained for 90% of the backbone amide protons (Figure 9). The overall rotational correlation time (*τ*_m_) of the ~13.8-KD Brd2 BD2 is 10 ns (as estimated from the average values of T_1_/T_2_), suggesting that BD2 is mainly monomeric in solution. The average ^1^H-^15^N NOE value is 0.76 ± 0.09, indicating that most of the regions of Brd2 BD2 are relatively rigid, corresponding well with the narrow distribution of conformers in the calculated ensemble. Some residues in the ZA and BC loop have relatively lower ^1^H-^15^N NOE values and these two loops construct the ligand-binding groove of Brd2 BD2 (Figure 9) and [see Additional file 1].

## Discussion

BET family is a less characterized novel class of bromodomain-containing proteins [18]. In the present work, we have determined the solution structure of the second bromodomain of Brd2, which shares more sequence identity with other BET bromodomains than with bromodomans of other families (Figure 2). The structure of Brd2 BD2 consists of a previously characterized four-helix left-handed bundle, with additional helices in ZA loop. Compared with other published bromodomain structures, extensive structural variations are found in loop regions (ZA and BC loop); especially, in ZA loop there exists an uncommon helix *π*D that is also present in BET family members Brd2 BD1 and Brd4 BD2. We speculate that the specific recognition of H4-AcK12 may involve this helix *π*D. Recently published structure of hsBRG1 bromodomain reveals a *β*Z sheet insertion between helices *α*Z and *α*Z'. The *β*Z sheet is proposed to have a role in orienting an acetylated substrate by changing the flexibility of the ZA loop [46]. The observations of the uncommon helix *π*D insertion in Brd2 BD2 and the *β*Z sheet insertion in hsBRG1 demonstrate that different bromodomains must have sufficient structural diversities to support specific recognition of acetylation codes. More and more information appears about the important functions of proteins in the BET family; especially that it is observed that Brd2, as well as Bdf1 and Brd4, remains mitotically associated with chromatin [19-21]. Our study provides a structural basis to further understand the functions of proteins in the BET family.

Consistent with a former study [21], our NMR titration experiments show that Brd2 BD2 specifically binds to H4-AcK12 peptide. We acquired the K_D _value (2.9 mM) for this specific binding and determined the interaction to be dynamic in solution. Brd2 BD2 binds to H4-AcK8 peptide with much lower affinity and does not bind to H2B-AcK5 peptide or unacetylated peptide H4-U at all. While the full length Brd2 can bind to H2B acetylated at K5/K12 [21], Brd2 BD2 can not. One possibility is that Brd2 BD1 is responsible for this interaction. The binding interface of H4-AcK12 peptide on Brd2 BD2 is further identified by NMR titration experiments, which is a conserved hydrophobic and electroneutral cavity with a negative-charged collar formed by five aspartate residues (D330, D338, D341, D385 and D387). These aspartate residues exhibited significant chemical shift changes in Brd2 BD2 titration with H4-AcK12 peptide and served as a secondary binding site as in hsGCN5 bromodomain [11]. The N-terminal tail of histone H4 is positively charged; and coincidently, when the positively charged residues (Lys 8, Lys 16 and Arg 17) flanking the acetylated lysine were mutated to negatively charged residues, Brd2 BD2 no longer bound to this peptide (Table 2 and Figure 6). When another flanking residue Ala 15 on H4-AcK12 peptide was substituted with glycine, it induced high aggregation of Brd2 BD2 with an unknown mechanism. Thus, the residue Ala 15 may also have a role in the specific recognition of H4-AcK12 by Brd2 BD2. The complex structure of scGCN5 with H4-AcK16 peptide revealed a primary interaction provided by acetylated lysine and a secondary interaction came from K+2 and K+3 positions [13]. Our result of Brd2 BD2 not binding to unacetylated peptide H4-U also solidate the notion that an acetylated lysine is a determinant in bromodomain-ligand recognition. Residues Lys 8, Lys 16 and Arg 17 (corresponding to K-4, K+4 and K+5 positions) provide a secondary recognition, while the residue Ala 15 (corresponding to K+3 position) may also be involved in the interaction with Brd2 BD2. So the interaction mode for Brd2 BD2 with H4-AcK12 peptide may be different from that of scGCN5-H4-AcK16 complex, which is further supported by the observation of large sequence and structural variations in the ZA and BC loops between Brd2 BD2 and scGCN5 [see Additional file 5].

The H4-AcK12 peptide binding pocket on Brd2 BD2 is constructed of many conserved or type-conserved resides. To determine which is crucial for H4-AcK12 recognition, we designed a series of mutations in the binding cavity, including V329A, Y339F, Y339A and N382A in respect that all these corresponding residues in hsP/CAF, hsCBP or scGCN5 bromodomains have extensive contacts with their ligands. Especially, the highly conserved residue N382 may be involved in orienting the acetylated Lys 12 as its corresponding residue N407 in scGCN5 [13]. Mutant N382A was constructed to examine its effect in H4-AcK12 interaction. ^15^N-HSQC spectra revealed that when the conserved residue V329 or N382 is mutated to alanine, the Brd2 BD2 conformation is disrupted throughout ZA and BC loops, even extending to the helices B and C for N382A mutant. Neither mutant V329A nor mutant N382A binds to H4-AcK12 peptide. For Y339F and Y339A mutants, the recombinant proteins have converted into inclusion body when expressed in *E. coli *(data not shown). Thus these highly conserved residues may be more important in maintaining a proper conformation for ligand recognition. On the other hand, L334A or L336A mutant just mildly disturbs a few adjacent residues and most likely doesn't affect the conformation of the binding pocket. However, these two mutants bind to H4-AcK12 with much lower affinities compared with wild type Brd2 BD2. Mutation-induced function disruption may be the main reason for L334A and L336A mutants not binding to H4-AcK12 peptide. Both residues L334 and L336 are localized in helix *π*D (residue 331–335), and L334G335 forms a two amino acids insertion unique to Brd2 BD2 and other BET members. Our results definitely show that the two amino acids insertion and helix *π*D are implicated in Brd2 BD2 and H4-Ack12 peptide interaction. However, the complex structure of Brd2 BD2 with H4 AcK12 peptide is needed to completely understand the interaction mechanism.

The solution structure of Brd2 BD2 is monomeric, whereas the crystal structure of Brd2 BD1 is determined to be a homodimer [41]. No inter-molecular NOEs were observed in Brd2 BD2, which was consistent with monomeric state as indicated by gel-filtration and GST-pull down analysis (see methods). It is not surprising that Brd2 BD1 shares only 46% sequence identity with BD2 and the dimeric residues are just partially conserved in BD2. In fact, the equivalent BD1s or BD2s from different proteins of BET family share higher homologies than the non-equivalent BD1 and BD2 in the same protein. Although both Brd2 BD1 and BD2 specifically recognize the same acetylated histone H4 tail, it is reasonable to propose that they play distinctive roles in Brd2 functions. In the Brd2 containing coactivator complex, BD1 may be a dimeric module and interacts with TBP [33], while BD2 may mainly serve to bind to acetylated histones. It is also probable that Brd2 BD1 and BD2 interact with acetylated histones in different context. And multi-bromodomains could enhance the affinity for acetylated histones. An intriguing question immediately rises and waits to answer. How Brd2 BD1 and BD2 cooperate to regulate Brd2 functions, and thus modulate its transcriptional activation effect?

## Conclusion

Brd2 BD2 is monomeric in solution and interacts weakly and dynamically but specifically with H4-AcK12. The helix *π*D in the long ZA loop may be a common characteristic of BET bromodomains. The conserved hydrophobic cavity is found to serve as a primary binding site for H4-AcK12 as in other bromodomains. A negatively charged collar of the cavity serves as a secondary binding site for H4-AcK12. Actually, the flanking basic residues on H4-AcK12 are important for their mutual recognition. Brd2 BD1 and BD2 may cooperate to regulate Brd2 functions. The structure of Brd2 BD2 will help to further characterize BET family members.

## Methods

### Cloning, expression and purification of Brd2

The DNA fragment encoding residues 301–408 corresponding to the second bromodomain of Brd2 was amplified from human brain cDNA library (Clontech) by a polymerase chain reaction (PCR) using the two primers 5'-CTCGAGATCTGGCATCTTGGCATAACGG-3' and 5'-CATATGGAACAGTTAAAACATTGCAATG-3' (restriction sites are underlined) designed based on the mRNA sequence of Brd2 [GenBank: M80613]. The reaction product (about 330 bp) was purified and then cloned into pGEM-T vector (Promega). The positive clones were identified by restriction digest. Then the DNA fragment was ligated into the *Nde*I/*Xho*I-cleaved plasmid pET22b(+) (Novagen), yielding plasmid pET22b-BD2. The plasmid pET22b-BD2 was then transformed into *Escherichia coli *BL21 (DE3) host cells for expression. Uniformly labeled recombinant Brd2 BD2 was produced using SV40 medium containing 0.5 g/l 99% ^15^N ammonium chloride and 2.5 g/l 99% ^13^C-glucose as the sole nitrogen and carbon source, respectively. Recombinant Brd2 BD2 was purified using Ni-chelating column (Qiagen). The purified recombinant Brd2 BD2 protein contains a C-terminal His tag (LEHHHHHH), with the N-terminal Met cleaved during expression (data from Mass spectrum). The purity of recombinant Brd2 BD2 was confirmed by Tricine-SDS-PAGE (15%, w/v) and the concentration was measured with BCA kits (Pierce). The ^15^N-labeled and ^13^C, ^15^N- labeled Brd2 BD2 were about 0.5–1.0 mM. All the samples for NMR contained 50 mM phosphate buffer (pH 5.8), 50 mM NaCl, 1 mM DTT, 1 mM EDTA in 90% H_2_O/10% D_2_O or in 99.96% D_2_O. Brd2 BD2 mutants were generated by conventional PCR method using pET22b-BD2 plasmid as template. Mutants V329A, L334A, L336A and N382A represented mutations of Val 329 to Ala, Leu 334 to Ala, Leu 336 to Ala and Asn 382 to Ala respectively. The uniformly ^15^N-labled proteins of Brd2 BD2 mutants were produced as described above. The integrity of Brd2 BD2 mutants were assessed by their ^15^N-HSQC spectra.

For GST fusion Brd2 BD2, the DNA fragment was cloned into pGEX-4T (Amersham Bioscience) resulting plasmid pGEX-BD2. The construct was also expressed in *Escherichia coli *BL21 (DE3). The GST fusion protein was purified using Sepharose 4B (Amersham Bioscience) and following the protocols.

### Characterization of Brd2 BD2 in solution

The state of purified recombinant His-tagged Brd2 BD2 in solution was analyzed using gel filtration chromatography and GST-pull down experiments. The gel filtration analysis was performed with a ÄKTA FPLC instrument (Amersham Biosciences) equipped with Hiload 16/60 Superdex 75 prep grade column. The purified His-tag fusion Brd2 BD2 was loaded onto the column equilibrated with a buffer containing 50 mM Tris and 500 mM NaCl (pH7.8) and was eluted with the same buffer. The molecular weight was calibrated with marker proteins included Cytochrome C (MW 12300), Myoglobin (horse, MW 17800), Chymotrypsinogen (MW, 25000), Albumin (egg, MW 45000), Albumin (bovine serum, MW 67000). In the analysis, Brd2 BD2 was eluted as a single peak, demonstrating the homogeneity of the sample. The apparent molecular weight of Brd2 BD2 was estimated to be 11 KD, corresponding well with the theoretic molecular weight of Brd2 BD2 (13.8 KD) (data not shown).

In GST-pull down experiments, the purified recombinant GST and GST-BD2 were immobilized on 200 *μ*l Sepharose 4B beads. After washing four times (1 ml every time) with GST binding buffer, the beads were then incubated with excessive amount of His-tag fusion Brd2 BD2 in total volume of 1 ml at 4°C overnight. The beads were washed by GST binding buffer supplemented with different concentrations of NaCl. Finally the proteins were eluted from beads by boiling. All samples were analyzed on Tricine-SDS-PAGE (15%, w/v) and stained with Coomassie brilliant blue. The GST-pull down experiments exhibited that the His-tagged and GST-tagged Brd2 BD2 didn't associate with each other in solution (data not shown). Accordingly, the recombinant Brd2 BD2 was homogenous and mainly monomeric in solution. Hence we moved forward to perform NMR experiments of Brd2 BD2.

### NMR spectroscopy and data processing

The NMR experiments were performed on a Bruker DMX600 spectrometer with self-shielded z-axis gradients. The following spectra were recorded at 298 K to obtain backbone and side chain resonance assignments: 2D ^1^H, ^15^N-HSQC, 2D ^1^H, ^13^C-HSQC, 3D triple-resonance spectra HNCO, HN(CA)CO, CBCA(CO)NH, CBCANH, C(CO)NH-TOCSY, H(CCO)NH-TOCSY, ^15^N-TOCSY, HBHA(CBCACO)NH, 2D CB(CC)HD-COSY (aromatics), 3D ^15^N-separted NOESY (mixing time 110 ms). The ^13^C/^15^N-labeled sample was then lyophilized and dissolved in 99.96% D_2_O, which was followed immediately with HSQC experiments to monitor the disappearance of NH signals at 293 K. After all of the peaks vanished, 3D HCCH-TOCSY, HCCH-COSY and ^13^C-separated NOESY (mixing time 130 ms) were recorded on this sample at 298 K.

NMR data processing was carried out using NMRPipe and NMRDraw software, and the data were analyzed with SPARKY. All software was run on a Linux system. Linear prediction was used to improve spectral resolution in the indirect dimensions where constant-time acquisition was used.

### Experimental restraints and NMR structure determination

NMR distance restraints were collected from two different NOESY spectra: 3D ^15^N-separated NOESY in H_2_O for amide protons, 3D ^13^C-separated NOESY in D_2_O for aliphatic protons. NOE restraints were grouped into four distance ranges: strong, 1.8–3.0 Å and 1.8–3.5 Å; medium, 1.8–4.0 Å and 1.8–4.5 Å; weak, 1.8–5.0 Å; and very weak, 1.8–6.0 Å. Considering that the spin diffusion effect could be serious for aliphatic protons, a more conservative distance estimation was used for the 3D ^13^C-separated NOESY; therefore, most medium-range and long-range NOEs from this spectra were put into the weak or very weak groups. The 1.8 Å lower limits were imposed only implicitly by the van der Waals repulsion force. For methyl protons, nonstereospecifically assigned methylene protons, and aromatic ring protons, r ^-6 ^summation averages were applied. Based on the chemical shifts values, the program CSI and TALOS were used to identify the secondary structure elements. The derived secondary structures were converted into restraints on *ϕ *and *ψ *angles. Hydrogen bond restraints were obtained by identifying the slow exchange amide protons mainly in the regular secondary structures.

Structures were calculated using the program CNS v1.1, employing a simulated annealing protocol for torsion angle dynamics. For the initial rounds of structure calculations, only sequential, intraresidual, medium-range NOEs, unambiguous long-range NOEs and dihedral angle restraints were used. Later, all other long-range NOEs and hydrogen bonds were introduced in consecutive steps. Simple impulsion nonbonded interactions were used during structure calculation. At the final stage, 200 conformers were calculated; of these, 20 conformers with the lowest energy were selected. The coordinates of the 20 selected structures and the mean structure have been deposited in the Protein Data Bank [PDB: 2G4A]. The qualities of the structures were assessed with PROCHECK. All residues are located in allowed regions on Ramachandran plot apart from K217 due to paucity of inter-residual NOEs. Being unassigned, the C-terminal His tag had no NMR restraints, and they were excluded from structure calculation.

### Histone N-terminal tail peptides and NMR titration

The peptides of acetylated histone tails H2B-AcK5 (residues 1–12 of histone H2B, SDPA-AcK-SAPAPKK, where AcK is an N^*ε*^-acetyl-lysine), H4-AcK8 (residues 1–12 of histone H4, SGRGKGG-AcK-GLGK), H4-AcK12 (residues 7–17 of histone H4, GKGLG-AcK-GGAKR), H4-L10A (Leu 10 to Ala mutation of H4-AcK12, GKGAG-AcK-GGAKR), H4-L10G (Leu 10 to Gly mutation of H4-AcK12, GKGGG-AcK-GGAKR), H4-A15G (Ala 15 to Gly mutation of H4-AcK12, GKGLG-AcK-GGGKR), H4-acid (Lys 8 to Asp, Lys 16 to Asp and Arg 17 to Glu mutations of H4-AcK12, GDGLG-AcK-GGADE) and the unacetylated peptide H4-U (residues 7–17 of histone H4, GKGLGKGGAKR) were purchased from Sangon. All these compounds were used without additional purification. For binding titrations, the samples of ^15^N-labeled Brd2 BD2 and Brd2 BD2 mutants were prepared in phosphate buffer (pH 5.8) as mentioned above, with the concentrations between 0.2 mM and 1.0 mM. Solutions of the peptides H2B-AcK5, H4-AcK8, H4-AcK12, H4-L10A, H4-L10G, H4-A15G, H4-U and H4-acid were prepared at concentrations between 55 mM and 100 mM, under identical buffer conditions. The ^1^H and ^15^N resonance variations were followed at 298 K by collecting HSQC experiments. Combined chemical shift perturbation was calculated using this equation, Δδppm=(ΔδHN)2+(ΔδNαN)2
 MathType@MTEF@5@5@+=feaafiart1ev1aaatCvAUfKttLearuWrP9MDH5MBPbIqV92AaeXatLxBI9gBaebbnrfifHhDYfgasaacH8akY=wiFfYdH8Gipec8Eeeu0xXdbba9frFj0=OqFfea0dXdd9vqai=hGuQ8kuc9pgc9s8qqaq=dirpe0xb9q8qiLsFr0=vr0=vr0dc8meaabaqaciaacaGaaeqabaqabeGadaaakeaacqqHuoariiGacqWF0oazdaWgaaWcbaGaemiCaaNaemiCaaNaemyBa0gabeaakiabg2da9maakaaabaGaeiikaGIaeuiLdqKae8hTdq2aaSbaaSqaaiabdIeaijabd6eaobqabaGccqGGPaqkdaahaaWcbeqaaiabikdaYaaakiabgUcaRiabcIcaOiabfs5aejab=r7aKnaaBaaaleaacqWGobGtaeqaaOGae8xSde2aaSbaaSqaaiabd6eaobqabaGccqGGPaqkdaahaaWcbeqaaiabikdaYaaaaeqaaaaa@489D@, with a scaling factor (*α*_*N*_) of 0.17.

### ^15^N relaxation experiments of Brd2 BD2

The ^15^N relaxation experiments were performed at 298 K on a Bruker DMX500 spectrometer. ^15^N T_1 _and T_2 _relaxation rates with one second recycle delay were measured with eight relaxation delays (21.2, 61.4, 142, 242, 363, 523, 804, and 1210 ms) and six relaxation delays (17.6, 35.2, 52.8, 70.4, 105.6, and 140.8 ms) respectively. The spectra for measuring ^1^H-^15^N NOE were performed with a two seconds relaxation delay followed by a three seconds period of proton saturation. And the spectra acquired without proton saturation employed a five seconds relaxation delay. The data analysis and exponential curve fitting were processed using SPARKY.

## Abbreviations

BET, Bromodomain protein with Extra C-Terminal domain; Brd2, bromodomain containing 2; Brd2 BD1/BD2, the first/second bromodomain of Brd2; NOE, nuclear Overhauser effect; NOESY, nuclear Overhauser enhancement spectroscopy; COSY, correlated spectroscopy; TOCSY, total correlation spectroscopy; HSQC, heteronuclear single quantum correlation; RMSD, root mean square deviation.

## Authors' contributions

HDH designed and carried out the experiments, drafted and revised the manuscript; JHZ helped to conceive and carry out the structural experiments; WQS helped to draft and revise the manuscript; XSW participated in preparation of the figures and revised the manuscript; JWW participated in preparation of the figures; JHW designed the experiments, carried out the structural experiments and revised the manuscript; YYS designed the experiments and revised the manuscript. All authors have read and approved the final manuscript.

## Supplementary Material

Additional file 1 Plots of NOE restraints and RMSD. (**A**) Plot of the number of NOE restraints per residue used in the calculation of the Brd2 BD2 structure. (**B**) Plot of backbone atoms and all heavy atoms average RMSD values from the mean structure of the final ensemble of 20 structures *vs*. the sequence of Brd2 BD2. Click here for file

Additional file 2 Summary of sequential and medium-range NOE patterns. The data are derived from ^15^N-separated and ^13^C-separated NOESY spectra of Brd2 BD2. The thickness of the bars of sequential NOEs indicates the relative intensities of the corresponding cross-peaks in NOESY, and horizontal lines indicate the observation of medium-range NOEs between residue pairs. Filled circles denote the locations of slowly exchanging amide protons. The short bars at the bottom represent consensus CSI predictions from C*α*, C*β*, C', and H*α *chemical shifts; bars below the line mean an index of -1, while those above the line mean an index of +1. Four or more consecutive bars of -1 not interrupted by a bar of +1 indicate *α*-helix. Three or more consecutive bars of +1 not interrupted by a bar of -1 indicate *β*-strand. Click here for file

Additional file 3 Backbone superposition of the experiment-derived bromodomain structures. (**A**) Backbone superposition of the average, energy-minimized structures of Brd2 BD2 (grey) with hsGCN5 (cyan); (**B**) Brd2 BD2 (grey) with scGCN5 (yellow); (**C**) Brd2 BD2 (grey) with hsCBP (magenta); (**D**) Brd2 BD2 (grey) with hsP/CAF (purple); (**E**) Brd2 BD2 (grey) and the two bromodomains from TAF_II_250 (red and green); (**F**) Brd2 BD2 (grey) with hsBRG1 (orange). The figure was generated with MOLMOL. Click here for file

Additional file 4 Table summarization of Brd2 BD2 mutants. The integrity of Brd2 BD2 mutants including V329A, L334A, L336A and N382A, were assessed by their ^15^N-HSQC spectra. The table lists the statistics of amide resonances disturbed by mutations. Click here for file

Additional file 5 Structural variations in the ZA and BC loops. An enlarge view of the structural variations in the ZA and BC loops between Brd2 BD2 (grey) and scGCN5 (cyan). A stick model showed the H4-AcK16 peptide of the scGCN5 complex structure. Click here for file
