# Supplementary figures and images for: Solution structure of the second bromodomain of Brd2 and its specific interaction with acetylated histone tails
Source: BMC Struct Biol. 2007 Sep 12;7:57. doi: 10.1186/1472-6807-7-57 (PMC2065866; doi:10.1186/1472-6807-7-57)

**Additional file 1**

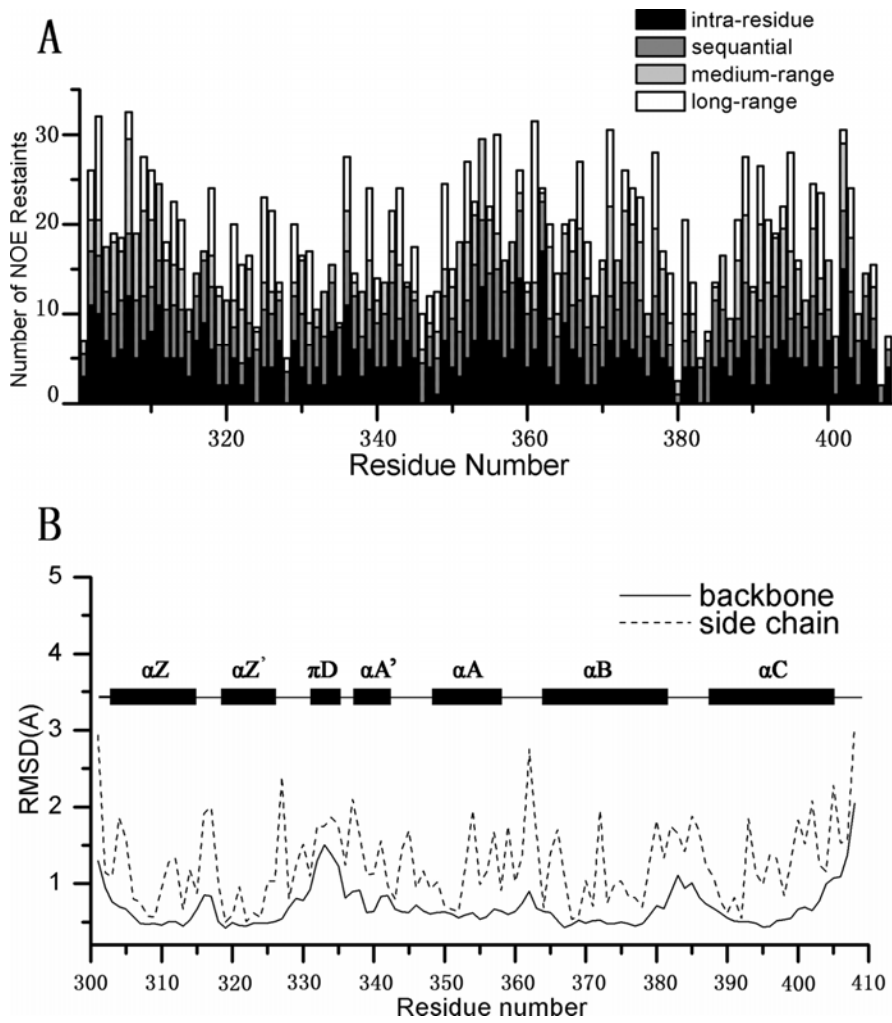

Supplement: Additional file 1 — Plots of NOE restraints and RMSD. (A) Plot of the number of NOE restraints per residue used in the calculation of the Brd2 BD2 structure. (B) Plot of backbone atoms and all heavy atoms average RMSD values from the mean structure of the final ensemble of 20 structures vs. the sequence of Brd2 BD2. [file 1472-6807-7-57-S1.pdf]

Additional file 2

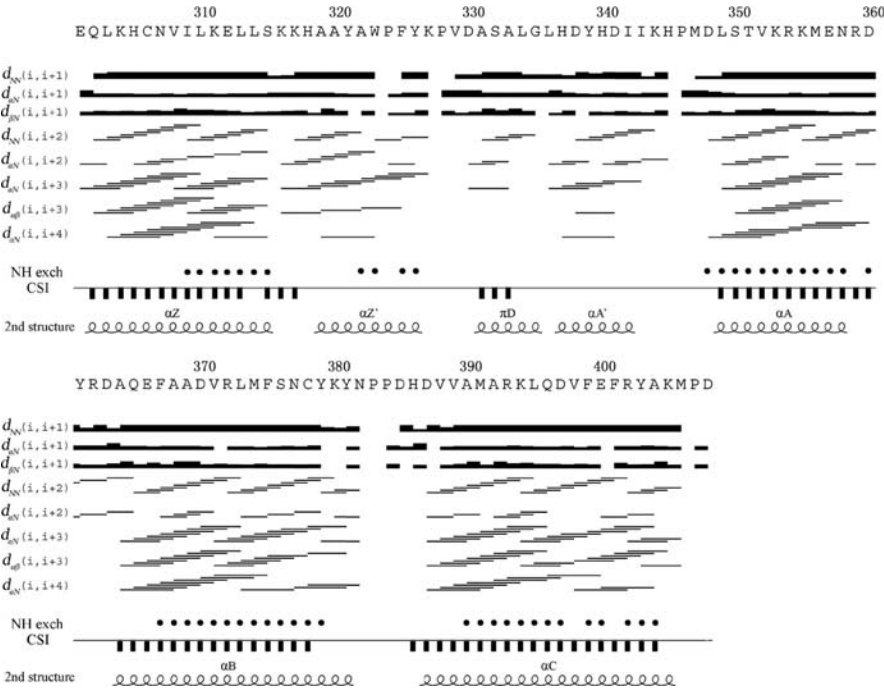

Supplement: Additional file 2 — Summary of sequential and medium-range NOE patterns. The data are derived from 15N-separated and 13C-separated NOESY spectra of Brd2 BD2. The thickness of the bars of sequential NOEs indicates the relative intensities of the corresponding cross-peaks in NOESY, and horizontal lines indicate the observation of medium-range NOEs between residue pairs. Filled circles denote the locations of slowly exchanging amide protons. The short bars at the bottom represent consensus CSI predictions from Cα, Cβ, C', and Hα chemical shifts; bars below the line mean an index of -1, while those above the line mean an index of +1. Four or more consecutive bars of -1 not interrupted by a bar of +1 indicate α-helix. Three or more consecutive bars of +1 not interrupted by a bar of -1 indicate β-strand. [file 1472-6807-7-57-S2.pdf]

**Additional file 3**

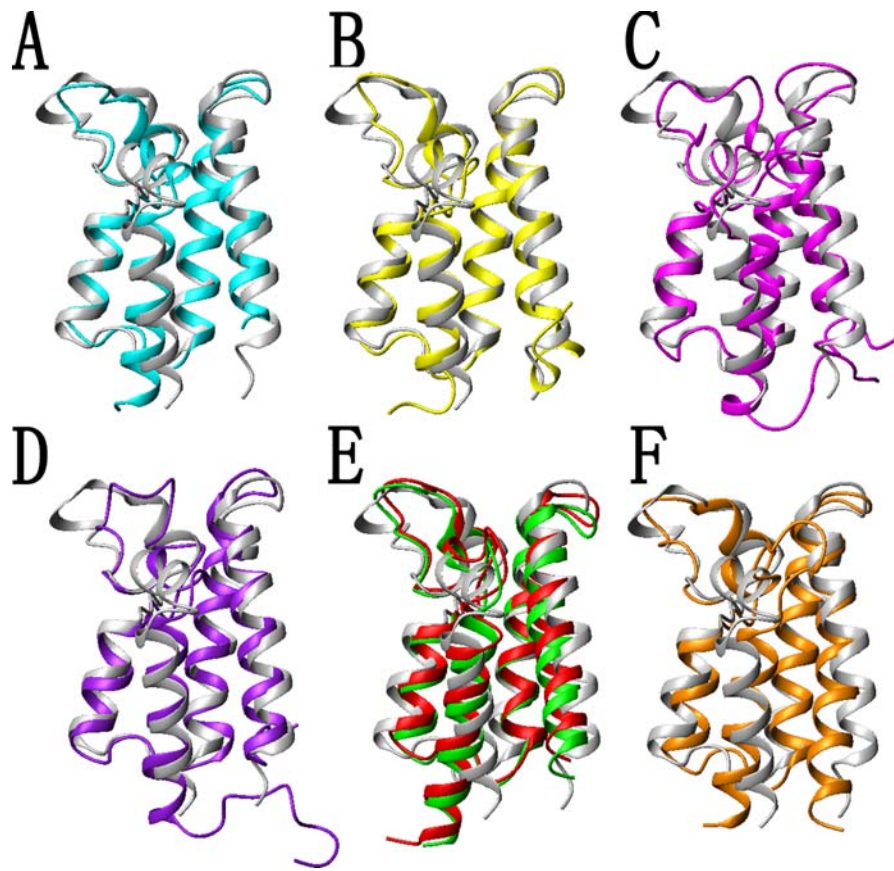

Supplement: Additional file 3 — Backbone superposition of the experiment-derived bromodomain structures. (A) Backbone superposition of the average, energy-minimized structures of Brd2 BD2 (grey) with hsGCN5 (cyan); (B) Brd2 BD2 (grey) with scGCN5 (yellow); (C) Brd2 BD2 (grey) with hsCBP (magenta); (D) Brd2 BD2 (grey) with hsP/CAF (purple); (E) Brd2 BD2 (grey) and the two bromodomains from TAFII250 (red and green); (F) Brd2 BD2 (grey) with hsBRG1 (orange). The figure was generated with MOLMOL. [file 1472-6807-7-57-S3.pdf]

**Additional file 5**

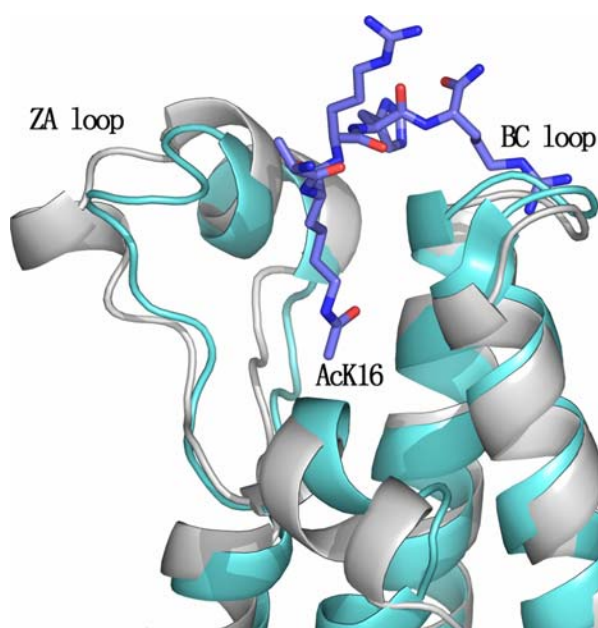

Supplement: Additional file 5 — Structural variations in the ZA and BC loops. An enlarge view of the structural variations in the ZA and BC loops between Brd2 BD2 (grey) and scGCN5 (cyan). A stick model showed the H4-AcK16 peptide of the scGCN5 complex structure. [file 1472-6807-7-57-S5.pdf]
