# Supplementary material for: Solution structure of the second bromodomain of Brd2 and its specific interaction with acetylated histone tails
Source: BMC Struct Biol. 2007 Sep 12;7:57. doi: 10.1186/1472-6807-7-57 (PMC2065866; doi:10.1186/1472-6807-7-57)
Supplement: Additional file 4 — Table summarization of Brd2 BD2 mutants. The integrity of Brd2 BD2 mutants including V329A, L334A, L336A and N382A, were assessed by their 15N-HSQC spectra. The table lists the statistics of amide resonances disturbed by mutations. [file 1472-6807-7-57-S4.pdf]

Additional file 4

Mutations of Brd2 BD2	Amide resonances disturbed from mutations
V329A	residues including W323 to M347 in the ZA loop, L349 and T351 in the amino terminus of helix αA, M374 to Y381 in the carboxy terminus of helix αB, N382 and D387 in the BC loop.
L334A	residues including D330 to D341
L336A	residues including K327 to M347 in the ZA loop, L349 and T351 in the amino terminus of helix αA, S376 and C378 to N382 in the carboxy terminus of helix αB and in the BC loop.
N382A	residues including W323 to M347 in the ZA loop, L349 and T351 in the amino terminus of helix αA, R372 to F399 comprised the carboxy terminus of helix αB, the BC loop and the amino half of helix αC.
